# The association between adverse life events and body weight change: results of a prospective cohort study

**DOI:** 10.1186/1471-2458-13-957

**Published:** 2013-10-14

**Authors:** Karin I Proper, H Susan J Picavet, Rik P Bogers, WM Monique Verschuren, Wanda JE Bemelmans

**Affiliations:** 1 Centre for Prevention and Health Services Research, National Institute for Public Health and the Environment (RIVM), P.O. Box 1, 3720 Bilthoven, BA, the Netherlands

**Keywords:** Life events, Body weight, Overweight, Prospective study, Stressful events

## Abstract

**Background:**

Stress has been shown to be a determinant of weight change and risk for obesity. To date, there is scarce evidence that stressful life events and their severity contribute to changes in body weight. We studied the association between the occurrence, impact of and adaptation to adverse life events and weight change and the role of initial weight status.

**Methods:**

Analyses were based on data from a population-based cohort of 2789 adults. Adverse life events, their impact and adaptation were measured retrospectively after baseline and follow-up weight and height measurements.

**Results:**

Over six years, participants gained an average of 2.8 kg. There were no differences in weight change between those who had experienced an adverse life event versus those who had not. However, the impact of life events had a significant interaction with initial weight status. Adults with a healthy weight showed an average weight reduction of 0.2 kg (95% CIs: -0.7 - 0.2), and overweight adults showed an average weight gain of 0.4 kg (95% CIs: -0.3 - 1.1) for each point increase in impact after experiencing an adverse life event. Further, a slower adaptation to events was significantly associated with greater weight loss among those who lost weight.

**Conclusions:**

We found no proof for an association between life events and weight change in the entire study sample, but we found that adults at a healthy weight responded differently to adverse life events than those who were overweight.

## Background

To prevent undesirable weight change and weight-related mortality and morbidity, it is necessary to have insight into factors that influence weight changes. Stress is one factor, since it has shown to be an important determinant for weight change and the risk for obesity. Several mechanisms have been suggested to explain this stress-related effect including physiological and behavioral processes [1,2]. For the behavioral pathway, Laugero et al. (2010) showed that people who perceived greater stress had lower participation levels in physical activity and a lower intake of fruit and vegetables, but a higher intake of salty snacks and sweets [2]. Previous research has shown that stress can induce eating [3-5], however, it can also suppress food intake [3,5,6]. Differences in reactions to stress leading to variations in impact on body weight may exist between obese and healthy weight adults or between men and women [3-5]. Based on three prospective cohorts, it was shown that the effect of stress on body weight differed according to baseline body mass index (BMI); positive associations were only found among overweight individuals [7-10]. In addition, Harding et al. also found differences in the relationships between psychosocial stress including life events and weight change by baseline BMI over a period of five years, emphasizing the role of initial weight status [11]. Further, Barry and Petry (2008) showed that overweight and obese women experienced more stressful life events than healthy weight women, whereas for men, obesity (but not overweight) was associated with more stressful life events [8]. In this context, it is noteworthy to refer to the appraisal of life events as opposed to the life events itself, which has been reported as one of the explanations why women experience more often life events prior to the onset of depression than men [12-14].

**Figure 1:**
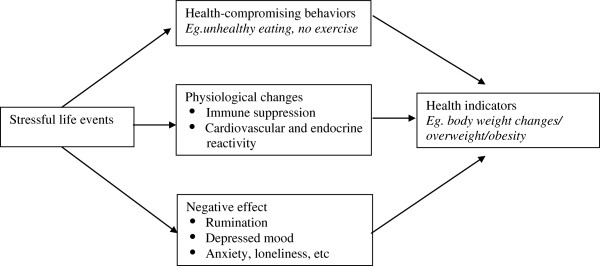
Conceptual model linking the experience of life events to body weight changes (based on model presented by Schwarzer, 2003).

Although there is no agreement about the definition of stress, it is clear that stress leads to poor health [15]. Stress can namely be defined from the biomedical sciences in which stress is a person’s response to adverse stimulation, whereas in psychology, stress is usually understood as the process where a person and the environment interact [15]. One of the theoretical approaches that link stress to life events is the stimulus-based approach of stress [16]. That theory treats life changes or life events as the stressor to which a person corresponds, and treats stress as synonymous with life events by the definition “life events are stress that require adaptation efforts”. The central proposition of this model is further that too many life changes in a relatively short period increase one’s vulnerability to illness [8]. In his book chapter, Schwarzer presented a conceptual model explaining three different pathways that link stressful life events to health indicators, including weight change (Figure 1) [15]. Life events can however also be conceptualized as a trigger for change or reinvention as shown by work of Ogden et al. [17,18]. In a qualitative study, they showed that sustained behavior change had been triggered by a significant life crisis if certain conditions were met [18]. These findings were supported using quantitative data with successful dieters reporting a higher number of life events than unsuccessful dieters [17]. These studies thus suggest that life events can promote behavior change that is facilitated by a reduced choice over their unhealthy behaviors and the belief that the behavioral changes are effective [17].

Previous studies on life events are generally cross-sectional and findings are mixed. Overall, some studies showed associations between life events and body weight in adults [7-10,19], whereas others found no relationship between stressful life events and BMI among African American women [20]. The studies included different life events, or a single life event (i.e. marital status change) and different study populations, which may explain the contrasting results [8]. Only one prospective cohort study focused on the relationship between the number of life events and weight change [9]. Within that study, several subgroups of adults who experienced many life events showed a gain in body mass after one year, but this effect disappeared after another year in most subgroups with the exception of men who tried to lose weight by dieting [9]. To date, there is thus little evidence that stressful life events contribute to changes in body weight. Moreover, following the model of Schwarzer [15], certain characteristics of life events, like the severity play a role in the effect on health-compromising behaviors and subsequently health indicators (e.g. body weight change). We hypothesized that severe events yield greater effects than events that are less severe.

Using prospective population-based data on adults, we investigated the association of the number, self-perceived impact of and adaptation to an adverse event with weight change.

## Methods

### Study design

Data came from the baseline and first follow-up measurement of the Doetinchem Cohort Study [21], which is part of the European Prospective Investigation into Cancer and Nutrition (EPIC) [21]. The origin of the Doetinchem Cohort Study lies within the Monitoring Project on Cardiovascular Disease Risk Factors (MP-CVDRF) carried out in three Dutch towns (Amsterdam, Doetinchem and Maastricht). From each of the three towns, about 12,000 men and women were examined. In the subsequent Monitoring Project on Chronic Disease Risk Factors (MORGEN-project), carried out from 1993–97, due to its number of inhabitants, in Doetinchem, only respondents from the MP-CVDRF were invited to participate, while in Amsterdam and Maastricht again random samples from the general population were examined [21]. Those from Doetinchem are thus in a prospective cohort (Doetinchem Cohort Study), measured every 5 years.

The participants’ weight and height were measured and they completed questionnaires on personal characteristics and lifestyle behaviors twice (1987-1991 and 1993-1997) within a six-year interval. After the second measurement, between 1996 and 1999, the participants completed an additional questionnaire, the Health and Life Experiences Questionnaire (HLEQ), which included questions on adverse life events. Ethical approval for the Doetinchem study was obtained by the Medical Ethical Committee TNO, Zeist, the Netherlands. All participants gave written informed consent and the study was approved according to the Helsinki Declaration guidelines.

### Adverse life events

The HLEQ was specifically developed for use in a prospective cohort among adults in Norfolk as part of EPIC [22-24].

For 17 predefined and one “other” adverse life events, participants could indicate if they had experienced such an event and in which year the event occurred. For events (n = 11) that could occur more than once, the participant was asked to report on the most recent occurrence and the most immediate preceding occurrence. Events related to health (e.g. serious illness or death), job (e.g. retired), or interpersonal situations (e.g. divorce, problem with friend or relative) (see also Table 1). For the present study, only the adverse life events that occurred between (but not including) the years of baseline data collection (1987-1991) and the first follow-up (1993-1997) were taken into account. Participants also specified how much the event had upset them on a four-point scale (from “not at all” (1) to “extremely” (4)) and to what extent they recovered from the event, also on a four-point scale (from “completely” (1) to “not at all” (4)). The higher the impact and adaptation score, the more the event upset the participant and the more slowly the participant recovered from the event, respectively.

**Table 1:** Description of life events and weight change per event that occurred between baseline and follow-up

	**Men (n = 1315)**				**Women (n = 1474)**			
	**n**	**Impact**^**a**^	**Adapt**^**b**^	**Mean/absolute weight change (kg)**	**n**	**Impact**^**a**^	**Adapt**^**b**^	**Mean/absolute weight change (kg)**
Serious illness	42	2.8	-^c^	**2.4/4.4**	44	2.9	- ^c^	**4.3/5.4**
Previous event	3	3.0	- ^c^		6	3.7	- ^c^	
Serious injury	27	2.4	- ^c^	**3.0/4.0**	30	2.9	- ^c^	**5.8/6.8**
Previous event	2	2.0	- ^c^		3	2.7	- ^c^	
Close relative with serious illness/injury	216	**2.8**	- ^c^	2.8/4.1	289	**3.3**	- ^c^	3.0/4.2
Previous event	32	**2.7**	- ^c^		40	**3.4**	- ^c^	
Separation or divorce	53	3.0	1.5	2.3/4.2	59	3.2	1.6	3.1/5.1
Previous event	2	2.5	1.0		6	3.7	2.2	
Serious problem with close friend/relative/neighbor	117	**2.7**	1.7	3.1/4.3	162	**3.1**	1.7	3.2/4.5
Previous event	10	2.9	1.6		16	3.1	1.8	
Retired from work	42	1.5	1.2	2.0/3.8	12	1.6	1.1	1.1/3.7
Previous event	1	1.0	1.0		0	-	-	
Partner retired from work	6	1.5	1.2	4.2/4.3	27	1.2	1.4	2.0/3.7
Previous event	1	1.0	1.0		0	-	-	
Termination of pregnancy^d^	6	2.3	1.4	3.9/5.0	12	3.1	1.8	8.4/9.1
Made redundant or sacked (fired)^d^	81	2.6	1.3	3.3/4.7	61	2.8	1.3	3.4/5.5
Partner made redundant or sacked (fired)^d^	33	2.0	1.2	2.9/4.3	58	2.8	1.2	2.9/4.9
Problems with police involving court appearance^d^	20	**2.3**	**1.3**	3.6/4.2	2	**4.0**	**2.5**	5.6/5.6
Partner problems with police involving court appearance^d^	2	1.5	1.0	4.2/4.2	8	2.5	1.4	5.6/6.7
Partner/spouse died	8	3.5	2.0	0.9/2.4	33	3.7	2.1	1.3/3.4
Previous event	0	-	-		0	-	-	
Son or daughter died	5	3.8	2.4	1.3/5.2	5	4.0	2.6	2.3/3.0
Previous event	0	-	-		1	4.0	2.0	
Father died	135	**2.7**	1.5	2.5/4.1	151	**3.1**	1.6	3.5/4.4
Mother died	108	**2.8**	**1.4**	2.7/3.6	123	**3.3**	**1.7**	2.6/3.9
Brothers/sisters died	68	**2.8**	**1.5**	**1.9/3.4**	64	**3.5**	**2.0**	**3.5/4.2**
Previous event	2	**2.0**	**1.0**		7	**3.7**	**2.0**	
Other unpleasant/disappointing event	171	**3.1**	**1.7**	2.6/4.1	237	**3.4**	**1.9**	3.1/4.3
Previous event	12	2.9	1.4		24	3.2	1.7	

Numbers that are bold indicate a significant difference between men and women in highest impact of the event, highest adaptation to the event, or mean weight change (*p* < 0.05).

^a^1 = not at all, 2 = a little, 3 = moderately, 4 = extremely.

^b^1 = completely, 2 = mostly, 3 = a little, 4 = not at all.

^c^Not asked for adaptation to the event.

^d^Not asked for previous event.

### Study population

**Figure 2:**
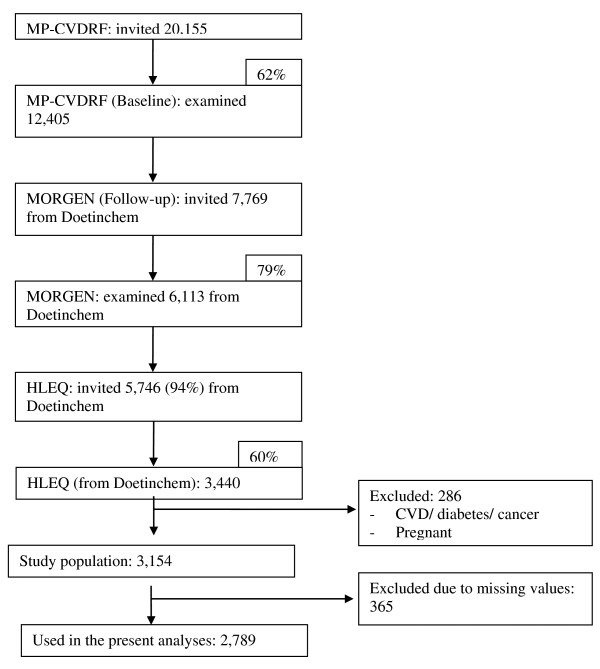
Flow chart of the inclusion process.

Figure 2 presents a flow diagram of the study population. An age- and gender-stratified random sample (n = 20,155) of men and women living in Doetinchem, aged 20–59 years, was invited to participate. Due to extension of the protocol, not all 12,405 participants in the MP-CVDRF could be re-invited. Instead, a random sample of in total 7,769 (response: 62%) of the respondents at baseline was invited to participate in the first follow-up after six years. A random sample of 7,769 adults from the 12,405 baseline articipants was invited.

From this group, 6,113 agreed to participate (response: 79%). The present study only included adults who had participated in both measurements and who had completed the HLEQ (n = 3,440). We excluded prevalent cases of cardiovascular diseases, cancer or diabetes, women who were pregnant at baseline or follow-up (n = 286), and those with missing or invalid data for key variables of the HLEQ for the analyses (n = 365). The final study population for the present study thus included 2,789 participants.

### Body weight

Trained staff measured the participants’ body weight and height. Body weight was measured to the nearest 100 g on calibrated scales [21]. BMI was calculated as body weight in kilograms divided by height in meters squared. Healthy weight was defined as a BMI < 25 kg/m^2^; overweight, including obesity, were defined as a BMI ≥ 25 kg/m^2^.

### Covariates

The variables used for adjustment or stratification in the statistical analyses, i.e. age, gender, educational level, and smoking cessation or initiation between baseline and follow-up, were assessed by questionnaire. Educational level (in three categories) was included as a covariate because of the expected variance in coping with adverse life events [25-27]. Time (years) between completing the follow-up and the HLEQ measurement was also included as a covariate, because of the various time frames between completing the follow-up and HLEQ measurement and because the adaptation to an event depends on the time since the event occurred.

### Analysis

Analyses were performed using SAS version 9.2 (SAS Institute Inc., Cary, NC, USA). Descriptive analyses were performed to report the characteristics of the total study population, and distinguished those who had experienced an adverse life event and those who had not. Of the adverse events experienced by an individual, including both the most recent and the previous events, only the impact of the most upsetting event and only the adaptation to the event an individual recovered from most slowly were calculated. As a previous event may have a greater impact than the most recent event, both were counted.

Linear regression analyses were performed to determine the association between the number, the highest impact of and the slowest adaptation to the events with changes in mean body weight. Both univariate and multivariate analyses were performed. In the multivariate analyses, the three life event parameters were entered simultaneously. In the adjusted analyses, educational level, gender, age, smoking, and time between completing the follow-up and the HLEQ measurement were included. All analyses were also performed with absolute weight change value and 5% weight change (including weight gain and weight loss) as the outcomes. The latter was done to get insight into the association between life events and a considerable weight change, in this case defined as a change of at least 5% from baseline body weight.

Interaction was tested for initial weight status (BMI < 25 kg/m^2^ versus BMI ≥ 25 kg/m^2^) with life events (occurrence, impact, adaptation) as well as interaction between gender and life events. Since either weight gain or loss may follow an adverse life event, stratified analyses were performed for those adults who had gained and lost weight (>0 kg, ≤0 kg).

## Results

Table 2 presents the demographic and weight-related characteristics at baseline, the life events and weight change between baseline and follow-up. At baseline, 47% of the study population was men and the mean age was 38.9 (9.9) years. Weight change did not differ between those who had or had not experienced an adverse life event (Table 2). During the six years of follow-up, participants gained an average of 2.8 kg (SD 4.7) (Table 2).

**Table 2:** Characteristics at baseline and weight change from baseline to follow-up

	**Total study population (n = 2789)**	**Participants who reported an adverse life event (n = 1503)**	**Participants who did not report one or more adverse life events (n = 1286)**	***p***^**b**^
Age (mean, SD)	38.9 (9.8)	39.0 (9.9)	38.8 (9.7)	0.46
Gender (% men)	47%	45%	49%	0.03
Educational level:				0.17
- lower (%)	56%	54%	58%	
- middle (%)	24%	24%	24%	
- higher (%)	20%	22%	19%	
Current smoker (%)	32%	33%	31%	0.29
Number of life events (mean, SD)	1.0 (1.2)	1.8 (1.1)	-	-
Highest impact (mean, SD)	3.1 (0.8)^a^	3.1 (0.8)	-	-
Highest adaptation (mean, SD)	1.7 (0.8)^a^	1.7 (0.8)	-	-
BMI (kg/m^2^) (mean, SD)	24.2 (3.2)	24.2 (3.2)	24.3 (3.2)	0.39
Overweight (≥25 kg/m^2^) (%)	38%	37%	38%	0.60
Mean weight change (kg) (mean, SD)	2.8 (4.7)	2.9 (4.8)	2.7 (4.6)	0.24
Absolute weight change (kg) (mean, SD)	4.1 (3.6)	4.2 (3.8)	4.0 (3.5)	0.21
* >5% gain (mean kg,%)	39%	39%	38%	0.26
* -5 to 5% change (mean kg,%)	56%	54%	57%	
* >5% loss (mean kg,%)	6%	6%	6%	

^a^The average highest impact and adaptation scores were 'only’ calculated for the participants with an adverse life event.

^b^the p-value is derived from an independent *t*-test or Chi-square test to test the difference between those who reported an adverse life event versus those who did not report an adverse life event.

Table 1 presents each adverse life event, with the occurrence, highest impact and adaptation score for men and women. The most frequently occurring events were serious illness or the injury of a close relative (n = 505), the death of the participants’ parent/s (n = 517), serious problems with a close friend/relative/neighbor (n = 279), and 'other unpleasant/disappointing’ events (n = 408) (Table 1). For some events, women showed higher impact and adaptation scores than men did (Table 1, data in bold).

Table 3 presents the results of the various regression analyses for the total study population, those with a healthy weight and overweight, and for weight losers and weight gainers. In the total study population, no associations were observed between body weight change and the number of life events, the impact of the most upsetting event or their adaptation to the event with the longest aftermath (Table 3). Results were similar using the absolute weight change and a 5% weight change as outcomes (data not shown). Participants who experienced one or more adverse events lost or gained >5% weight (n = 6 and 39%, respectively) as often as those who experienced no events (n = 6 and 38%, respectively) (Table 2).

**Table 3:** Results of the linear regression analyses to the association between life events with body weight changes (n = 2789)

	**Mean weight change**			
	**Univariate**		**Multivariate**^**a**^	
	**β**	**95% CI**	**β**	**95% CI**
**Total study population (n = 2789)**				
Crude				
Number of life events experienced	0.10	-0.04, 0.25	0.11	-0.14, 0.37
Highest impact	0.06	-0.23, 0.36	0.16	-0.22, 0.54
Highest adaptation	-0.06	-0.39, 0.27	-0.17	-0.55, 0.20
Adjusted^b^				
Number of life events experienced	0.09	-0.05, 0.23	0.11	-0.14, 0.36
Highest impact	-0.09	-0.40, 0.21	0.02	-0.36, 0.40
Highest adaptation	-0.19	-0.52, 0.14	-0.23	-0.60, 0.13
**BMI <25 kg/m**^**2**^**(n = 1745)**				
Crude				
Number of life events experienced	0.09	-0.09, 0.26	0.20	-0.10, 0.49
Highest impact	-0.17	-0.52, 0.18	-0.17	-0.61, 0.27
Highest adaptation	-0.15	-0.55, 0.25	-0.16	-0.61, 0.28
Adjusted^b^				
Number of life events experienced	0.07	-0.10, 0.24	0.17	-0.13, 0.46
Highest impact	-0.25	-0.60, 0.11	-0.23	-0.67, 0.21
Highest adaptation	-0.26	-0.66, 0.14	-0.23	-0.67, 0.21
**BMI ≥25 kg/m**^**2**^**(n = 1044)**				
Crude				
Number of life events experienced	0.13	-0.13, 0.39	-0.01	-0.46, 0.44
Highest impact	0.41	-0.13, 0.95	0.67	-0.02, 1.37
Highest adaptation	0.09	-0.49, 0.66	-0.24	-0.91, 0.42
Adjusted^b^				
Number of life events experienced	0.15	-0.11, 0.40	0.09	-0.36, 0.53
Highest impact	0.15	-0.40, 0.70	0.36	-0.34, 1.05
Highest adaptation	-0.10	-0.67, 0.46	-0.30	-0.94, 0.35
**Weight losers (n = 705)**				
Crude				
Number of life events experienced	-0.11	-0.28, 0.06	-0.14	-0.42, 0.14
Highest impact	-0.14	-0.44, 0.16	0.07	-0.29, 0.45
Highest adaptation	-0.38*	-0.71, -0.05	-0.37	-0.75, 0.01
Adjusted^b^				
Number of life events experienced	-0.07	-0.24, 0.10	-0.07	-0.35, 0.22
Highest impact	-0.13	-0.44, 0.19	0.11	-0.28, 0.50
Highest adaptation	-0.37*	-0.71, -0.03	-0.40*	-0.78, -0.01
**Weight gainers (n = 2084)**				
Crude				
Number of life events experienced	0.07	-0.06, 0.20	-0.04	-0.27, 0.20
Highest impact	-0.03	-0.32, 0.25	-0.02	-0.38, 0.34
Highest adaptation	0.19	-0.12, 0.51	0.22	-0.14, 0.57
Adjusted^b^				
Number of life events experienced	0.08	-0.05, 0.21	-0.001	-0.22, 0.22
Highest impact	-0.18	-0.47, 0.10	-0.17	-0.53, 0.19
Highest adaptation	0.06	-0.24, 0.37	0.13	-0.21, 0.48

*Abbreviations*: *ß* Beta coefficient, *CI* Confidence interval, *BMI* Body Mass Index.

*Statistically significant at *p* < 0.05.

^a^Number of life events, highest impact and highest adaptation were included in the same model.

^b^Adjusted for age, gender, smoking cessation, smoking initiation, educational level, and interval between completion of the follow-up measurement and the HLEQ.

There was a statistically significant interaction between initial weight status and the mean highest impact (beta = 0.70, SE = 0.34, *p* = 0.04). In adults with a healthy weight, a one-point higher impact score (on a four-point scale) was associated with a non-significant body weight reduction of 0.2 kg (95% CIs: -0.7 - 0.2). In overweight adults, a one-point higher impact score was associated with a non-significant weight gain of 0.4 kg (95% CIs: -0.3 - 1.1). No interaction was observed for gender and adverse life events (Table 4).

**Table 4:** Results of the linear regression analyses to the interaction between gender and life events (n = 2789)

	**β**	**SE**	**P**
Number of events *gender	-0.16	0.11	0.16
Highest impact of events*gender	-0.05	0.04	0.09
Highest adaptation to events* gender	-0.03	0.34	0.93

The stratified analyses for weight losers and weight gainers revealed that in participants who lost weight, a slower adaptation to events was significantly associated with more weight loss (0.4 kg per one-point increase in highest adaptation).

## Discussion

To the best of our knowledge, this study is the first examining the association between adverse life events and body weight change, that also included the perceived severity of and adaptation to the event, and studied the role of initial weight status and gender in this association.

The absence of an association between adverse life events and body weight change tends to confirm other studies showing that the effect vanishes with time. Rookus et al. (1988) showed an increase in BMI in some subgroups of young adults one year after an adverse life event, but most effects had disappeared after the following year [28]. These findings suggest that if stressful life events affect weight change they might only have a short-term effect thus explaining our null results. This lack of a “long-term effect” might also be explained by a weak long-term impact of life events that involve acute stress on lifestyle or physiological processes. Our study seems to be mainly based on events that do not systematically induce chronic stress or impact body weight. The life events under study generally referred to crisis occurrences and not to daily chronic stressors, like hardship and work conditions. Although it is plausible that hardship situations generate more weight change than a single adverse stressful event, for some single occurrences, like being diagnosed with a serious disease or the loss of a loved one, long-term effects can be observed.

Another study found contrasting body weight changes during the study period [6]. They showed a decrease in body weight between a pre-crisis (i.e. in the year prior to the crisis) and the crisis (i.e. one month after the event), and an increase in body weight at the post-crisis measurement (six months after the crisis) [6].

Other studies showing a relation between life events and weight change explained the direction of the weight change by the type of the event and its impact on the behavioral choice and function [17-19]. For example, weight loss due to a life event was perceived as more positive and as reducing the choice and function of food and increasing the choice and function over exercise [17,19].

The interaction found in our and other studies [2,4] between initial weight status and impact of an adverse life event may support the assumption that overweight adults lose control more often over their eating behavior after an adverse life event compared to adults with a (sustained) healthy body weight. If so, this is of relevance and supports the attention for a psychological component in behavioral weight loss interventions.

Previous studies have shown that the association between weight gain and stress or adverse life events differed between men and women [7,10,22]. In the present study, there were gender differences since women reported a higher impact and slower adaptation to some of the adverse events, and also showed a greater weight change for the same event than men did. This was in line with Surtees and Wainwright (2007), who found that women reported similar events as more upsetting, and took longer to recover from the effects, than men [29].

Although we found no support in our data for a relationship between adverse life events and weight change, we think further research is warranted because there are several plausible mechanisms for this association. Previous research has shown that adults perceiving stress or other psychological problems have lower levels of physical activity and higher caloric intake or intake of sweets [2,6,30,31]. Considering those results and the null results found in our study on body weight change, it may be that life events resulted in unhealthier eating or lower levels of physical activity, but did not subsequently yield body weight changes. Despite physical activity and diet questions were included in the questionnaire, these do not yield appropriate information about the energy balance, which was a reason for not including them in the analyses as covariate. Moreover, the main question of this study was to explore whether life events are associated with weight change. The mechanism in this is however another, though interesting, question. A future study could thus be to examine the mechanisms, by a mediation analysis, which is possible even without a significant relationship between the independent and dependent variable [32].

The study strengths are the large study population, longitudinal design with six years of follow-up, objectively measured body weight and height, and the inclusion of a number of adverse life events. Furthermore, to the best of our knowledge, this is the first longitudinal study to also include the impact of and adaptation to adverse life events in relation to weight change. In doing so, we used the highest individual impact and adaptation score to account for the severity of the event. Therefore, we avoided the effect that events, which were most upsetting to the individual or took the most time to recover from, would be attenuated by the other (less severe) events. By taking the most severe events, greater changes in body weight were assumed compared to events that were less severe or took a shorter time to recover from. The downside of this selection is that the possible accumulation of the impact of the events was neglected. Since we also studied the association between the number of events and weight change, we believe this side effect was not present. Furthermore, we chose to examine the association of the impact of adverse events with body weight change as a continuous measure, because we believed this would be more sensitive to detect an association in a general population (i.e. not being overweight or obese). By using BMI categories, gradual and small changes in body weight that did not result in a change in BMI category could not be detected.

Some study limitations should also be acknowledged. First, although we adjusted for several variables that we considered as key potential covariates, other factors may also interact with lifestyle or body weight, (e.g. employment, social support from family). To explain, work can function as a compensation of the negative mood and thereby protect the individual from adverse responses to the life event. If so, adjustment for these factors would have attenuated the associations (if present). Second, we decided to exclude events that occurred before baseline measurements because of the possible influence on the participant’s initial weight. Although this impact was not calculated, we cannot rule out the possibility that adverse life events that occurred before baseline had an effect that carried over to the period between the two weight measurements. Third, although it is less likely that weight change precedes an event, our data could not determine if an adverse life event had preceded the weight change. Thus this study could not establish causality, because we don't know the chronology: the process preceding an adverse event, as well as the overall hardship in which adverse events are more frequent (e.g. diseases, general life long adversity) can both explain changes in lifestyle and/or BMI. It should also be considered that the measurement of adverse life events was retrospective, which may have implications for the results. Answers could have been influenced by the participant’s current health or social situation, and thereby the perceived severity of the events. However, since we assume that an equal distribution of events would have been perceived as less severe versus more severe, we believe this retrospective measurement did not lead to substantial recall bias. Finally, the generalizability of our findings is limited because of the non-responses. No information on the level of education, BMI or lifestyle behaviors was available from non-participants at baseline, but it is known that the less educated adults and those who smoked are underrepresented [21]. Respondents to the HLEQ reported higher socioeconomic status, better subjective health and healthier lifestyle behaviors than non-respondents [33]. These differences between non-responders and responders might have influenced the findings. For example, assuming that less educated adults use ineffective coping styles more often (e.g. through behavioral strategies), this under-representation of less educated adults may have led to an underestimation of the results.

## Conclusion

In conclusion, our findings showed no association between adverse life events with objectively measured weight change among adults. The findings suggest that when studying this relationship, initial weight status should be taken into account.

## Competing interests

The authors declare that they have no competing interests.

## Authors’ contributions

KP, RB, SP and WB participated in the design of the study and drafting the manuscript. SP and MV are project leader and PI, respectively of the cohort study and were thus involved in the data collection. KP and RB with the support of GD (see acknowledgement) performed the statistical analysis and interpreted the data. All authors have made substantial contributions to conception and revised the manuscript critically for important intellectual content. All authors read and approved the final manuscript.

## Pre-publication history

The pre-publication history for this paper can be accessed here:

http://www.biomedcentral.com/1471-2458/13/957/prepub
